# Myocarditis and colchicine: a new perspective from cardiac MRI

**DOI:** 10.1186/1532-429X-18-S1-O100

**Published:** 2016-01-27

**Authors:** Daniel Morgenstern, John Lisko, Nicholas C Boniface, Brandon M Mikolich, J Ronald Mikolich

**Affiliations:** 1 Sharon Regional Health System, Hermitage, PA USA; 2 Northeast Ohio Medical University, Rootstown, OH USA

## Background

Because of anatomic proximity, myocarditis is often associated with pericarditis. Although randomized trials have not delineated a successful evidence-based therapy for myocarditis, there is evidence of considerable benefit in treating pericarditis with a regimen of colchicine and a non-steroidal anti-inflammatory drug (NSAID). Cardiac MRI (CMR) is capable of detecting both myocarditis and pericarditis using T2 weighted pulse sequences and delayed enhancement imaging with gadolinium. This study was designed to assess the effect of a colchicine/NSAID treatment regimen for myocarditis identified by CMR.

## Methods

An institutional CMR database was queried for all patients with pericarditis who had associated myocarditis. Those patients with at least 1 follow-up CMR study to assess response to therapy with a colchicine/NSAID regimen constituted the study population. The follow-up CMR studies were categorized and tabulated as resolved, partially resolved or no improvement with respect to myocarditis, based on consensus of 2 experienced CMR interpreters.

## Results

Forty-eight patients in the database were found to have myocarditis/myo-pericarditis by CMR and at least 1 follow-up CMR study. Twenty-seven of these patients received colchicine and 17(63%) showed complete resolution of myocarditis on their last CMR study. Of the 21 patients who did not receive colchicine, only 8(38%) had resolution of myocarditis by CMR study.

## Conclusions

Randomized trials for treatment of myocarditis were done prior to the availability of CMR imaging for this diagnosis, and were mainly comprised of severe cases presenting as heart failure. Myocarditis appears more readily diagnosed with CMR, especially in its less fulminant presentations. This study suggests that myocarditis, documented by CMR, is responsive to colchicine, with rates of successful therapy similar to those of published reports of colchicine for pericarditis. These data also suggest that CMR imaging should be considered in any future randomized trials of myocarditis therapy, including colchicine.

**Figure 1:**
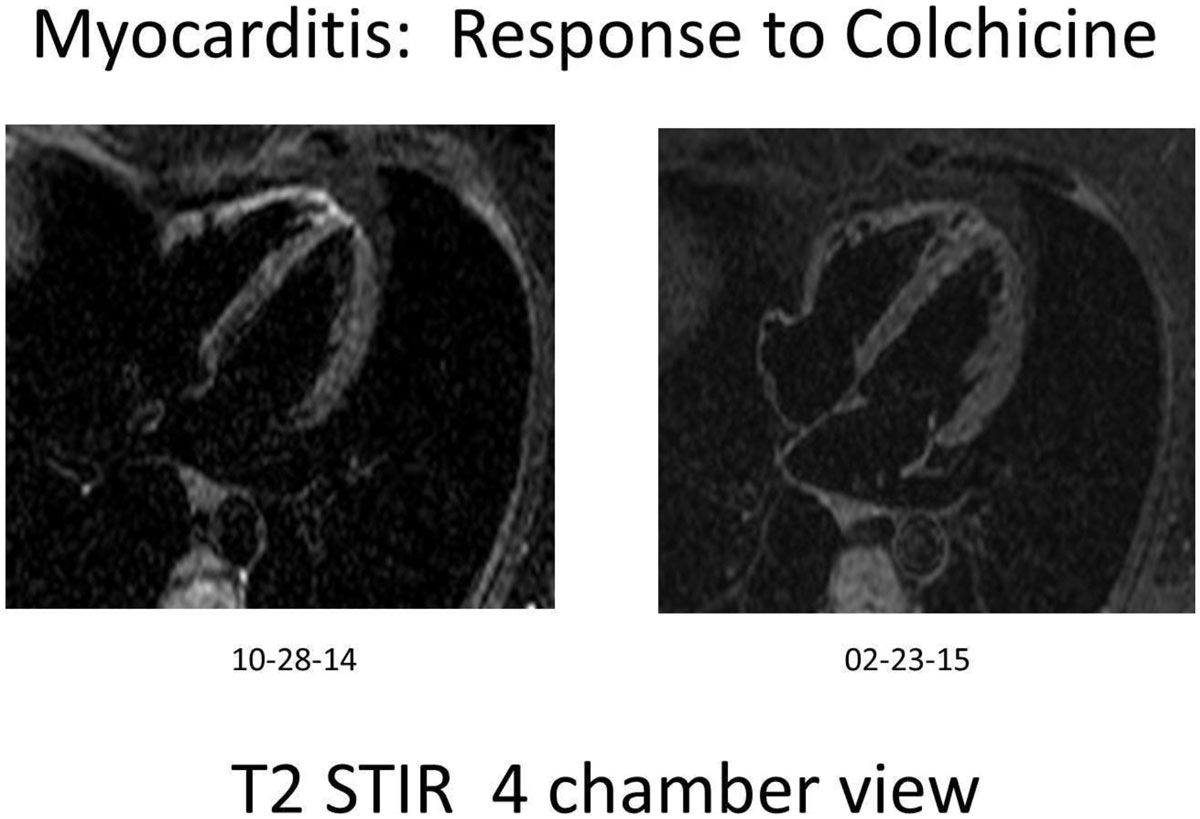
**Myocarditis response to colchicine therapy**.

